# Percutaneous transhepatic choledochoscopic lithotripsy for bile duct stones in elderly patients: a case report and technical summary

**DOI:** 10.3389/fmed.2025.1672408

**Published:** 2025-12-11

**Authors:** Pan Liu, Shun-Hai Liu, Song Zhang, Xin Xiang

**Affiliations:** 1 Department of Hepatobiliary Surgery, The First People's Hospital of Neijiang, Neijiang, Sichuan, China; 2 Department of Hepatobiliary Surgery, The Sixth People's Hospital of Chengdu, Chengdu, Sichuan, China

**Keywords:** hepatolithiasis, choledocholithiasis, PTCD, PTCSL, CBDs

## Abstract

**Background:**

The clinical management of elderly patients with complex intra- and extrahepatic bile duct stones and a history of multiple abdominal surgeries is often challenging. The development of PTCSL offers a novel therapeutic option for this population.

**Case summary:**

An 82-year-old male with extensive, complex hepatolithiasis and concomitant choledocholithiasis was transferred to our institution for definitive treatment following PTCD placement at another hospital. The patient had undergone three prior abdominal surgeries (open cholecystectomy, choledocholithotomy, and partial enterectomy) and coronary stent implantation for coronary artery stenosis 6 months prior. PTCSL was successfully performed to completely clear the biliary calculi. No stone recurrence was observed during 1-year follow-up. Integrating contemporary literature with the authors’ technical experience, we summarize critical procedural insights to advance clinical implementation of this technique.

**Conclusion:**

Two-step PTCSL represents a safe and effective therapeutic option for elderly patients with complex intra- and extrahepatic bile duct stones and multiple high-risk factors. Comprehensive preoperative planning combined with judicious utilization of surgical instruments can significantly enhance procedural efficacy and safety.

## Introduction

Biliary stone disease is relatively prevalent in East Asian countries, with surgical intervention serving as the primary therapeutic approach. Conventional procedures include common bile duct exploration (CBDE), endoscopic retrograde cholangiopancreatography (ERCP), and hepatic resection (1). However, the management of refractory cases—particularly in patients with a history of multiple biliary surgeries or alimentary tract reconstruction—poses significant challenges due to the disease’s high recurrence rate and complex stone distribution. These factors increase surgical difficulty and elevate postoperative complication rates (2, 3). Elderly patients with prior operations face particularly unfavorable outcomes, as many exhibit strong aversion to repeat open surgery.

The evolution of minimally invasive techniques has led to the development and refinement of percutaneous choledochoscopic lithotripsy (PTCSL). Current evidence supports its efficacy in treating intrahepatic and extrahepatic bile duct stones (4–6). This report details the successful application of PTCSL in an elderly patient with complex hepatolithiasis and choledocholithiasis and a history of multiple abdominal surgeries. Integrating contemporary literature with the authors’ technical experience, we summarize critical procedural insights to advance clinical implementation of this technique.

## Case presentation

**Figure 1:**
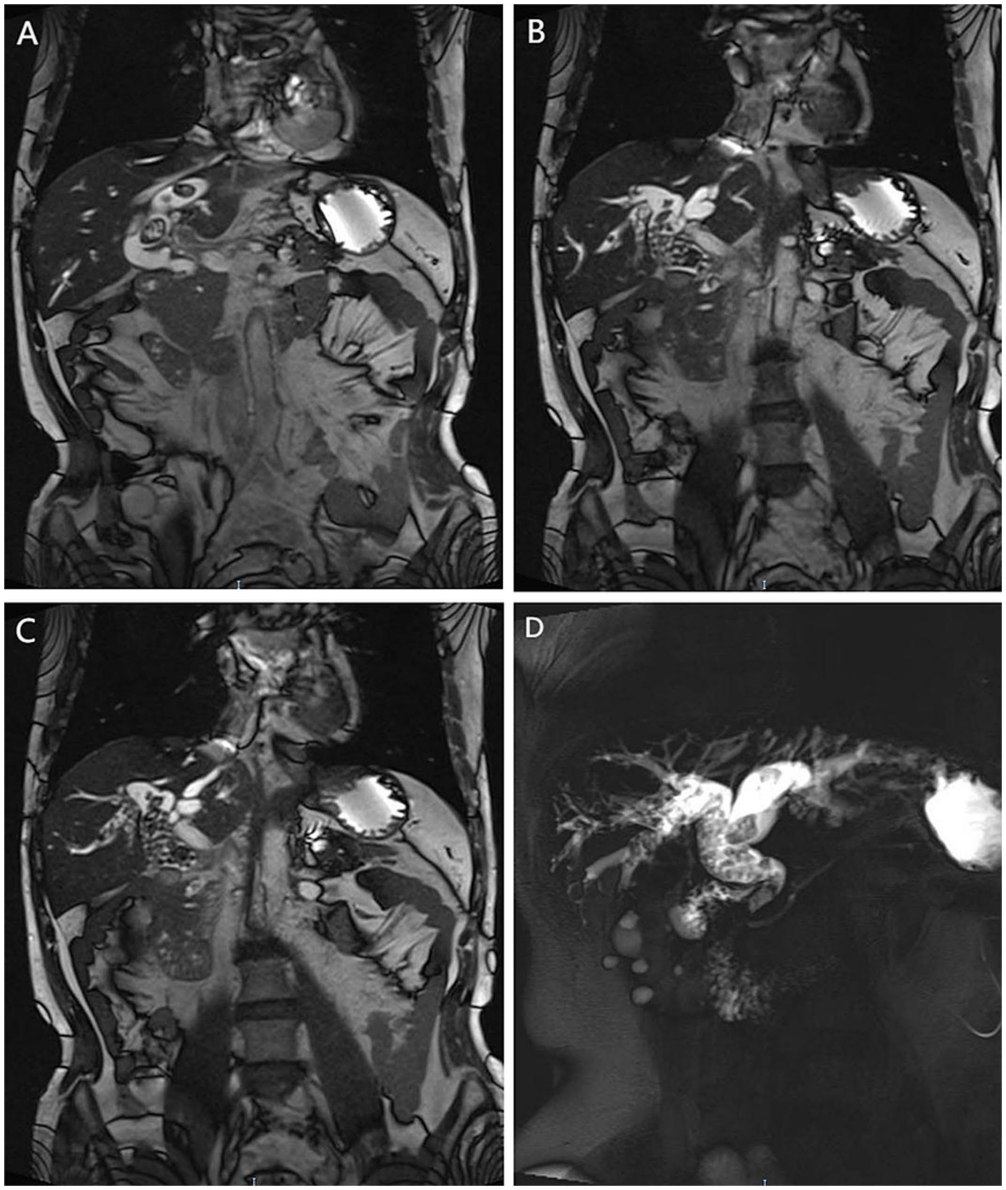
Biliary stone distribution visualized by MR/MRCP. **(A)** Coronal MR image: Stones in the left hepatic duct; **(B,C)** Coronal MR images: Stones in the right hepatic duct and common bile duct; **(D)** MRCP image: Overall morphology of the biliary tract and stone distribution.

**Figure 2:**
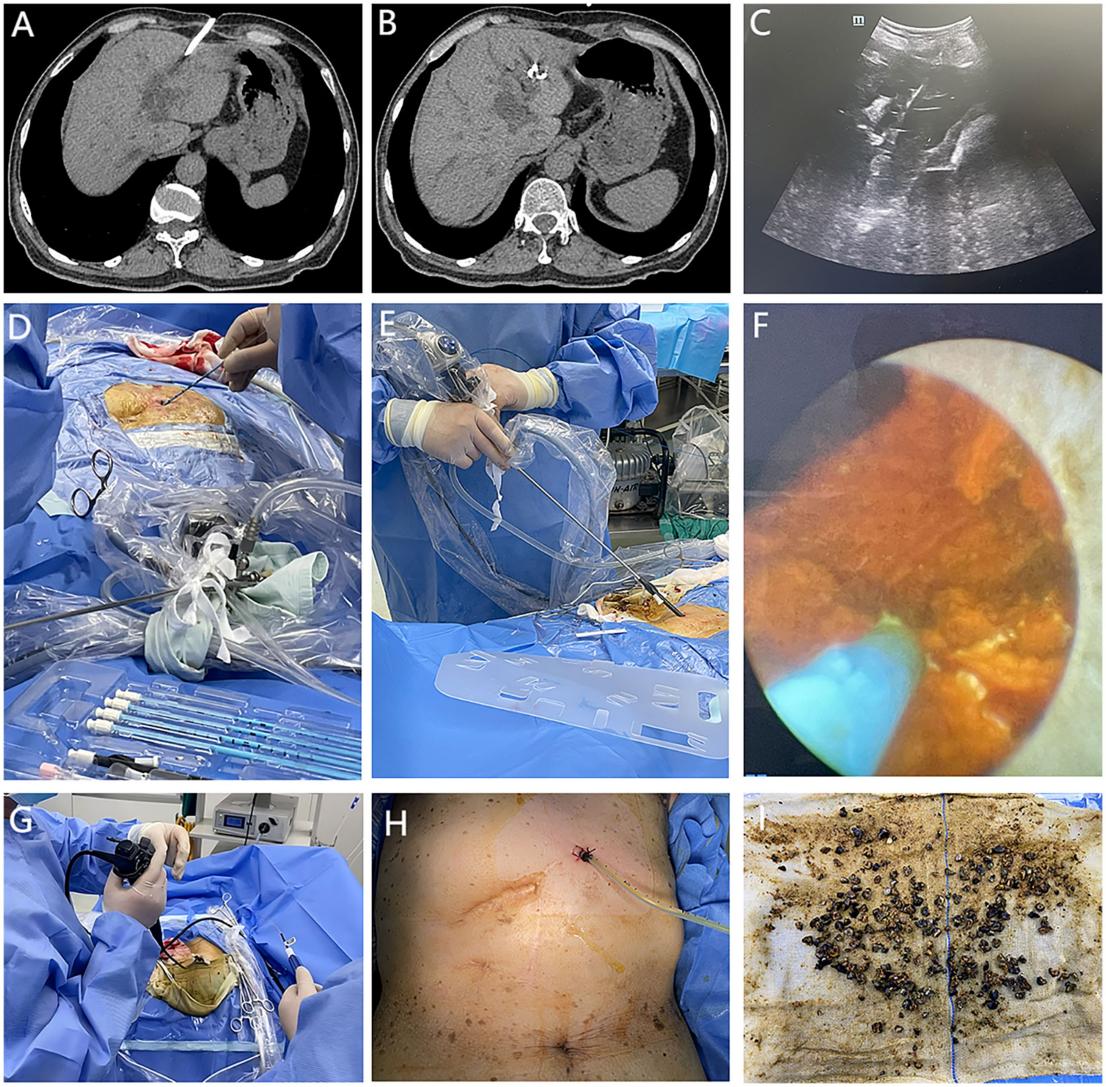
**(A,B)** Non-contrast CT images showing the positional relationship between the PTCD catheter and intrahepatic bile ducts; **(C–I)** Sequential visualization of the procedural steps for PTCSL.

An 82-year-old male was admitted for definitive management of hepatolithiasis and choledocholithiasis following percutaneous transhepatic cholangiodrainage (PTCD), having presented to a local hospital 1 month prior with acute symptoms of chills, high fever, jaundice, and hypotension. Diagnostic evaluation at the referring institution confirmed multiple intrahepatic and common bile duct stones (CBDs) with suppurative cholangitis, necessitating emergent PTCD placement; subsequent clinical improvement permitted discharge with an indwelling catheter and referral to our center. His surgical history included three abdominal procedures—open cholecystectomy (19 years ago), CBDE for stone extraction (9 years ago), and partial small bowel resection for intestinal obstruction (4 years ago)—supplemented by coronary stenting 6 months before admission for critical stenosis, requiring ongoing anticoagulation therapy. Due to 1 month of PTCD drainage and subsequent medical therapy, the patient’s liver function was classified as Child-Pugh class A at admission. Laboratory tests revealed no significant abnormalities, including complete blood count, coagulation profile, renal function tests, amylase, and tumor markers. While abdominal MRI/MRCP identified multiple calculi in the left hepatic duct, right posterior sectional duct, and common bile duct (Figure 1), and concurrent CT imaging confirmed PTCD catheter placement in the segment III bile duct (Figures 2A,B).

## Treatment and surgical procedure

**Figure 3:**
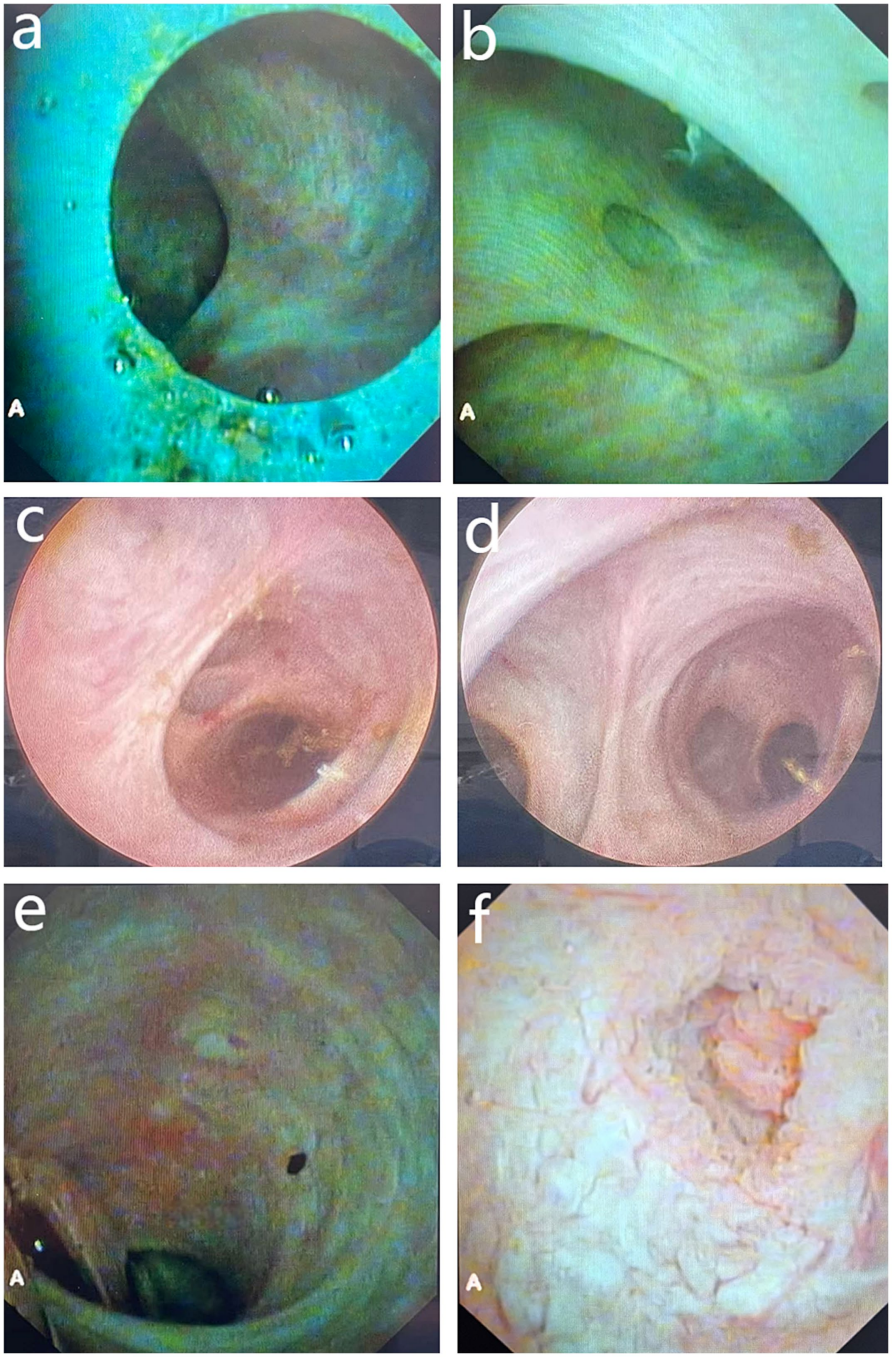
Post-clearance re-choledochoscopy. **(a)** Segment III bile duct; **(b)** segment II bile duct; **(c)** right anterior sectional duct; **(d)** right posterior sectional duct; **(e)** common bile duct; **(f)** duodenal papilla. Images **(a,b,e,f)** were obtained using a conventional flexible choledochoscope; images **(c,d)** were obtained using a rigid choledochoscope.

Low-molecular-weight heparin was substituted for oral anticoagulation perioperatively, and PTCD patency was maintained. Given the presence of both CBDs and extensively distributed intrahepatic stones, ERCP was deemed high-risk for potential procedural failure or significant prolongation of operative time due to technical challenges. Moreover, the preexisting PTCD tract, established 1 month earlier at another hospital, provided immediate percutaneous access to the biliary system, facilitating efficient tract formation for stone removal. Additionally, the planned PTCSL utilizing a rigid cholangioscope was expected to enhance operational flexibility and stone retrieval efficiency. Based on these considerations and after detailed discussion with the patient regarding treatment options, PTCSL was jointly selected as the definitive intervention. PTCSL was performed 1 week after admission under general anesthesia (the total indwelling time of the PTCD tube was 5 weeks). The procedure involved the following steps:

1. Preoperative planning and catheter exchange: based on preoperative imaging, the skin entry point and trajectory to the intrahepatic bile duct were mapped. Ultrasound-guided wire placement into the bile duct was achieved, followed by PTCD catheter removal (Figures 2A–C).
2. Tract dilation: after skin incision, the sinus tract was sequentially dilated over the guidewire using a minimally invasive dilation system, ultimately positioning an 18F sheath within the bile duct (Figure 2D).
3. Cholangioscopic lithotomy: a 9.5Fr rigid ureteroscope (serving as a cholangioscope) was advanced through the pre-placed sheath for direct visualization of biliary stones. Smaller stones were flushed out through the sheath under pressurized saline irrigation. Larger stones were fragmented with holmium laser lithotripsy before removal. Stones with a maximum diameter smaller than the inner diameter of the sheath could be easily flushed out; if resistant to irrigation, foreign body forceps or stone extraction baskets were employed adjunctively. For biliary branches inaccessible to the rigid cholangioscope, a 16Fr flexible cholangioscope (or a 3.6Fr single-use ultra-slim cholangioscope if necessary) was used in conjunction with extraction baskets for visualization and stone retrieval. (Figures 2E–G).
4. Comprehensive ductal evaluation: post-lithotomy, both rigid and flexible cholangioscopy confirmed complete stone clearance and direct visualization of all biliary branches, including those initially identified as stone-bearing, and ultrasonography was again performed to confirm the absence of residual stones within the liver. (Figure 3).
5. Drainage placement: a guidewire was positioned in the common bile duct via flexible cholangioscopy, followed by placement of a drainage catheter (Figure 2H). The procedure concluded with external drainage and stone collection (Figure 2I).

## Postoperative management and follow-up

The patient received postoperative antibiotics and fluid therapy with open drainage for 2 days prior to discharge. The catheter was clamped incrementally over 1 week and removed after confirmatory outpatient cholangiography demonstrated biliary patency. Long-term ursodeoxycholic acid therapy was initiated, with regular follow-up advised. No recurrence was observed during 1-year surveillance.

## Discussion

This patient represents a paradigmatic case of surgically challenging biliary stone disease, characterized by advanced age, multiple abdominal surgical histories, severe acute presentation, complex stone distribution, and anticoagulation-associated coagulopathy, compounded by marked aversion to repeat laparotomy.

During acute cholangitis, PTCD achieves rapid biliary decompression. When combined with pharmacotherapy, this intervention mitigates sepsis progression and multiorgan dysfunction, creating critical opportunities for systemic recovery (7). However, significant external bile diversion remains a limitation. The integration of ultrasound-guided cholangiography for initial PTCD placement in two-step PTCSL enables real-time ductal mapping and precise catheter positioning (8). Our procedural experience confirms that securing the catheter distal to the common bile duct or traversing strictures during first step establishes effective drainage, permitting extended PTCD clamping to minimize bile loss. Crucially, retrospective analyses demonstrate reduced intraoperative hemorrhage with two-step versus single-step PTCSL—attributed to tract maturation from initial PTCD placement, which diminishes hemorrhagic events and prevents biliary peritonitis (9, 10). Consequently, we advocate two-step PTCSL for high-risk patients, particularly the elderly, with ultrasound-cholangiography-guided PTCD placement recommended when clinically feasible.

Furthermore, optimizing preoperative planning and surgical instrument selection is crucial for enhancing initial and ultimate stone clearance rates while reducing operative time in this procedure. A retrospective study by Yong-Qing Ye et al. (11) (*n* = 140 single-step PTCSL patients) demonstrated that preoperative DynaCT for 3D biliary imaging significantly improved initial stone clearance (88.6% vs. 27.1%, *p* = 0.000), stricture resolution (97.1% vs. 78.6%, *p* = 0.001), and reduced complications (8.6% vs. 41.4%, *p* = 0.000) compared to no reconstruction, highlighting its importance. Intraoperative ultrasound is now essential for single-step PTCSL and valuable for assessing clearance in two-step PTCSL (12, 13). Common choledochoscopes include rigid, conventional flexible, and disposable ultra-thin flexible types (6). Rigid scopes offer ease of use, clear vision, large workspace, and high efficiency, accessing some dilated level III ducts, but risk duct injury with difficult angles. Conventional flexible scopes have better maneuverability for moderate angles but poorer level III access and operability. Ultra-thin scopes provide excellent level III access for improved clearance and stricture detection, though lower efficiency increases time and cost (14). Based on our experience, preoperative imaging should guide puncture site selection to maximize rigid scope advantages, supplemented by flexible or ultra-thin scopes as needed for comprehensive visualization. Stones are extracted using pneumatic/holmium laser lithotripsy with pressurized saline irrigation (4, 6). For complex stone distributions, consider dual or multiple tracts (15).

Regarding the prevention of postoperative stone recurrence, multiple factors influence recurrence, but biliary strictures are the most critical determinant (6, 10). Literature reports indicate that the incidence of biliary strictures accompanying hepatolithiasis ranges from 42.0 to 75.0% (16), with biliary-enteric anastomotic strictures occurring in approximately 11.9% of cases (17). Following complete stone clearance, the management of biliary strictures is paramount for preventing stone recurrence. Current modalities for addressing strictures include rigid dilatation under choledochoscopic guidance, balloon dilation, electrosurgical incision, and prolonged stenting with biliary stents or drainage tubes (6, 18). Consequently, definitive management of biliary strictures must be addressed following PTCSL to achieve the goal of preventing stone recurrence.

Finally, it should be emphasized that for elderly patients with multiple CBDs and extensively distributed intrahepatic stones, as reported in this case, although PTCSL was successfully performed to achieve complete stone clearance, it is undeniable that the continuous advancement of ERCP combined with SpyGlass technology (19) and endoscopic ultrasound (20, 21) has led to rapid developments in both treatment and recurrence prevention strategies for complex hepatolithiasis. However, it is noteworthy that no direct comparative studies have been published to date between PTCSL and the other two guided modalities for stone extraction. Future head-to-head comparisons among these techniques are warranted to better elucidate the indications, advantages, and limitations of each therapeutic approach.

In conclusion, two-step PTCSL represents a safe and effective therapeutic option for elderly patients with complex intra- and extrahepatic bile duct stones and multiple high-risk factors. Comprehensive preoperative planning combined with judicious utilization of surgical instruments can significantly enhance procedural efficacy and safety.

## Data Availability

The original contributions presented in the study are included in the article/Supplementary material, further inquiries can be directed to the corresponding author.
