# Supplementary material for: Percutaneous transhepatic choledochoscopic lithotripsy for bile duct stones in elderly patients: a case report and technical summary
Source: Front Med (Lausanne). 2025 Dec 11;12:1672408. doi: 10.3389/fmed.2025.1672408 (PMC12738867; doi:10.3389/fmed.2025.1672408)
Supplement: Supplementary file 1 [file Data_Sheet_1.pdf]

CARE
case report guidelines

CARE Checklist of information to include when writing a case report

(cc) BY-NC-ND

Topic	Item	Checklist item description	Reported on Line
Title	1	The diagnosis or intervention of primary focus followed by the words "case report"	Line 2 / Page 2
Key Words	2	2 to 5 key words that identify diagnoses or interventions in this case report, including "case report"	Line 21 / Page 2
Abstract (no references)	3a	Introduction: What is unique about this case and what does it add to the scientific literature?	Lines 2-17 / Page 3
	3b	Main symptoms and/or important clinical findings	Lines 20-26 / Page 3
	3c	The main diagnoses, therapeutic interventions, and outcomes	Lines 4-7 / Page 4
	3d	Conclusion—What is the main "take-away" lesson(s) from this case?	Lines 13-15 / Page 3
Introduction	4	One or two paragraphs summarizing why this case is unique (**may include references**)	Lines 19-20 / Page 7
Patient Information	5a	De-identified patient specific information.	Line 20 / Page 3
	5b	Primary concerns and symptoms of the patient.	Lines 20-26 / Page 3
	5c	Medical, family, and psycho-social history including relevant genetic information	Lines 26-29 / Page 3
	5d	Relevant past interventions with outcomes	Lines 20-29 / Page 3
Clinical Findings	6	Describe significant physical examination (PE) and important clinical findings.	Lines 1-7 / Page 4
Timeline	7	Historical and current information from this episode of care organized as a timeline	Not Provided
Diagnostic Assessment	8a	Diagnostic testing (such as PE, laboratory testing, imaging, surveys).	Lines 1-7 / Page 4
	8b	Diagnostic challenges (such as access to testing, financial, or cultural)	Not Applicable
	8c	Diagnosis (including other diagnoses considered)	Lines 4-7 / Page 4
	8d	Prognosis (such as staging in oncology) where applicable	Lines 4-7 / Page 4
Therapeutic Intervention	9a	Types of therapeutic intervention (such as pharmacologic, surgical, preventive, self-care)	Lines 9-19 / Page 4
	9b	Administration of therapeutic intervention (such as dosage, strength, duration)	Lines 19-29 / Page 4 Lines 1-20 / Page 5
	9c	Changes in therapeutic intervention (with rationale)	Not Applicable
Follow-up and Outcomes	10a	Clinician and patient-assessed outcomes (if available)	Lines 16-20 / Page 5
	10b	Important follow-up diagnostic and other test results	Lines 16-20 / Page 5
	10c	Intervention adherence and tolerability (How was this assessed?)	Not Applicable
	10d	Adverse and unanticipated events	Not Applicable
Discussion	11a	A scientific discussion of the strengths AND limitations associated with this case report	Lines 10-18 / Page 4
	11b	Discussion of the relevant medical literature **with references**.	Lines 27-29 / Page 5 Page 6 Lines 1-18 / Page 7
	11c	The scientific rationale for any conclusions (including assessment of possible causes)	Lines 11-13 / Page 3
	11d	The primary "take-away" lessons of this case report (without references) in a one paragraph conclusion	Lines 19-22 / Page 7
Patient Perspective	12	The patient should share their perspective in one to two paragraphs on the treatment(s) they received	Not Provided
Informed Consent	13	Did the patient give informed consent? Please provide if requested	Yes ☑ No ☐
